# Cost of hospital management of *Clostridium difficile* infection in United States—a meta-analysis and modelling study

**DOI:** 10.1186/s12879-016-1786-6

**Published:** 2016-08-25

**Authors:** Shanshan Zhang, Sarah Palazuelos-Munoz, Evelyn M. Balsells, Harish Nair, Ayman Chit, Moe H. Kyaw

**Affiliations:** 1 Usher Institute of Population Health Sciences and Informatics, University of Edinburgh, Medical School, Teviot Place, Edinburgh, EH8 9AG UK; 2 Department of Preventive Dentistry, Peking University School and Hospital of Stomatology, 22 Zhongguancun South Avenue, Beijing, 100081 China; 3 Sanofi Pasteur, Lyon, France; 4 Sanofi Pasteur, Swiftwater, PA USA; 5 Lesli Dan Faculty of Pharmacy, University of Toronto, Toronto, Ontario Canada

**Keywords:** *Clostridium Difficile*, Economic analysis, Systematic review, Meta-analysis

## Abstract

**Background:**

*Clostridium difficile* infection (CDI) is the leading cause of infectious nosocomial diarrhoea but the economic costs of CDI on healthcare systems in the US remain uncertain.

**Methods:**

We conducted a systematic search for published studies investigating the direct medical cost associated with CDI hospital management in the past 10 years (2005–2015) and included 42 studies to the final data analysis to estimate the financial impact of CDI in the US. We also conducted a meta-analysis of all costs using Monte Carlo simulation.

**Results:**

The average cost for CDI case management and average CDI-attributable costs per case were $42,316 (90 % CI: $39,886, $44,765) and $21,448 (90 % CI: $21,152, $21,744) in 2015 US dollars. Hospital-onset CDI-attributable cost per case was $34,157 (90 % CI: $33,134, $35,180), which was 1.5 times the cost of community-onset CDI ($20,095 [90 % CI: $4991, $35,204]). The average and incremental length of stay (LOS) for CDI inpatient treatment were 11.1 (90 % CI: 8.7–13.6) and 9.7 (90 % CI: 9.6–9.8) days respectively. Total annual CDI-attributable cost in the US is estimated US$6.3 (Range: $1.9–$7.0) billion. Total annual CDI hospital management required nearly 2.4 million days of inpatient stay.

**Conclusions:**

This review indicates that CDI places a significant financial burden on the US healthcare system. This review adds strong evidence to aid policy-making on adequate resource allocation to CDI prevention and treatment in the US. Future studies should focus on recurrent CDI, CDI in long-term care facilities and persons with comorbidities and indirect cost from a societal perspective. Health-economic studies for CDI preventive intervention are needed.

**Electronic supplementary material:**

The online version of this article (doi:10.1186/s12879-016-1786-6) contains supplementary material, which is available to authorized users.

## Background

*Clostridium difficile* is the leading cause of infectious nosocomial diarrhoea in the United States (US) [1] and the incidence and severity of *C. difficile* infection (CDI) are increasing [2]. CDI is associated with significant morbidity and mortality; it represents a large clinical burden due to the resultant diarrhoea and potentially life-threatening complications, including pseudomembranous colitis, toxic megacolon, perforations of the colon and sepsis [3–5]. Up to 25 % of patients suffer from a recurrence of CDI within 30 days of the initial infection. Patients at increased risk of CDI are those who are immuno-compromised, such as those with human immunodeficiency virus (HIV) or who are receiving chemotherapy [6–8], patients receiving broad-spectrum antibiotic therapy [9, 10] or gastric acid suppression therapy [9, 11], patients aged over 65 years [10], patients with serious underlying disease [12], patients in intensive care units (ICUs) [10], or patients who have recently undergone non-surgical gastrointestinal procedures or those being tube-fed [10].

CDI represents a significant economic burden on US healthcare systems. Infected patients have an increased length of hospital stay compared to uninfected patients, besides there are significant costs associated with treating recurrent infections. A few systematic reviews of cost-of-illness studies on CDI cost are available [13–21]. These reviews mainly listed the range of reported cost of their respective observation period or were limited by the small number of included studies or inadequate control for confounding factors. No meta-analysis of large number of cost data in the US has been conducted to date. The cost for patients discharged to long-term care facility (LTCF) and recurrent CDI management are understudied. The cost of case management and total financial burden of CDI treatment in the US is therefore underestimated and remains controversial.

The aim of the current study is to conduct a systematic review and meta-analysis of currently available data to identify and quantify the financial burden attributable to CDI, and to further estimate the total economic burden of CDI hospital management in the US.

## Methods

### Search strategy

English-language databases with online search tools were searched for to offer maximum coverage of the relevant literature: Medline (via the Ovid interface 1946 to July 2015); EMBASE (via the Ovid interface 1980 to July 2015); The Centre for Review and Dissemination Library (incorporating the DARE, NHS EED, and NHS HTA databases); The Cochrane Library (via the Wiley Online Library) and Health Technology Assessment Database (1989 to July 2015).

We supplemented our data by searching relevant published reports from: National epidemiological agencies, Google search for grey literature and hand searched the reference lists of the included studies. The general search headings identified were: Clostridium difficile, economic, costs, cost analysis, health care costs, length of stay, hospitalization. Examples of the strategy for Medline and EMBASE are listed in Additional file 1.

### Study selection

All studies that reported novel direct medical cost and/or indirect costs related to CDI management were included. Review articles, comments, editorials, letters, studies of outbreaks, case reports, posters and articles reporting results from economic modelling of a single treatment measure (i.e. cost effectiveness of faecal transplantation) were excluded in the final analysis. All relevant publications from January 2005 to July 2015 were included in the search. We included the following healthcare settings: hospitals, long-term care facilities and community. Geographical scope covered the US. We did not apply any language restriction. Our predefined inclusion and exclusion criteria are shown in Additional file 1.

### Data extraction

Two reviewers (SP, SZ) independently selected the included articles and extracted data. After combining their results, any discrepancies were solved by discussion with HN and MK.

The primary outcomes were CDI-related costs (total costs of those with CDI and other comorbidities) and CDI-attributable costs (total costs of CDI management only, after controlling for the confounders). For studies with control groups (e.g. matched patients without CDI), the CDI-attributable cost extracted was either the cost provided by the articles or calculated by reviewers using the CDI-related cost minus the treatment cost of control groups. The secondary outcome was resource utilization associated with CDI, i.e. CDI-related length of stay (LOS) in hospital and CDI-attributable LOS. The study characteristics of each article were extracted. These included basic publication information, study design, statistical methods, economic data reporting characteristics and population information.

When multiple cost data were presented in a study, we included only one cost estimate for each population subgroup as per the priority below:

- Matched data > Unmatched data.
- Adjusted model results > Unadjusted model results.
- Regression model results > Calculated difference.
- Total cost/charges > Subgroup cost/charge (i.e. survivors, died).
- Median (Interquantile Range: IQR) > Mean (Standard Deviation, SD).

All costs/charges data were inflated to 2015 US$ equivalent prices adjusted for the Consumer Price Index. If the price year was not reported, it was assumed to be the last year of the data collection period. In cases where charges were reported without cost-to-charge given, costs were estimated using a cost-to-charge ratio of 0.60, which is commonly used value in US health economic studies [22].

### Meta-analysis and estimation of national impact

We carried out meta-analysis for cost studies following a Monte Carlo simulation approach, as reported by Jha et al [23] and Zimlichman et al [17], bearing in mind the heterogeneity of the included studies. For each subgroup of CDI, we synthesized the data and reported a point estimate and 90 % confidence intervals (CIs) for the CDI-related cost, CDI-attributable cost and their respective LOS. For each included study, we simulated distribution with pooled results weighted by sample size. We fitted a triangular distribution for each of the included studies based on their reported measures of central tendency and dispersion, i.e. mean and 95 % CI, median and IQR, or median and range. Then we simulated 100,000 sample draws from the modeled distribution of each study. At each iteration, we calculated the weighted average of all included studies. Finally, we reported the mean and 90 % CI from the resulting distribution of the 100,000 weighted average of CDI. This approach facilitated the combination of cost data and eliminated the limitation of combining non-normally distributed data. Monte Carlo simulations were conducted using the Monte Carlo simulation software @RISK, version 7.0 (Palisade Corp).

**Fig. 1:**
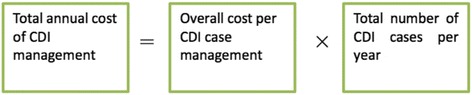
Formula for total annual cost calculation

We estimated the national financial impact of CDI on the US healthcare system, by determining the potential boundaries. The higher boundary was the total number of CDI cases in the US in 2011 extracted from Lessa et al [24], while the lower boundary was the result from a meta-analysis to estimate the total burden of CDI cases in the US [25] (For detailed results see Additional file 1). The total annual cost of CDI management was calculated multiplying the average cost of management per case of CDI, with the total number of CDI cases per year in the US (Fig. 1). We assumed that all CDI cases received treatment in hospital. A point estimate of the final cost (with range) was reported based on a Monte Carlo simulation of 100,000 sample draws.

### Sensitivity analysis

We extracted the total number of CDI patients and CDI-attributable costs from previous studies [25] and reviews [17, 26] to carry out a sensitivity analysis of our total cost estimates.

### Quality assessment

**Fig. 2:**
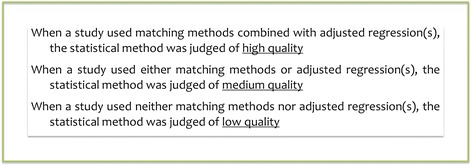
Quality Assessment Method

The quality of the studies included was assessed mainly based on the complexity of the statistical method (Fig. 2). All studies were included in the final analyses.

## Results

### Search results

**Fig. 3:**
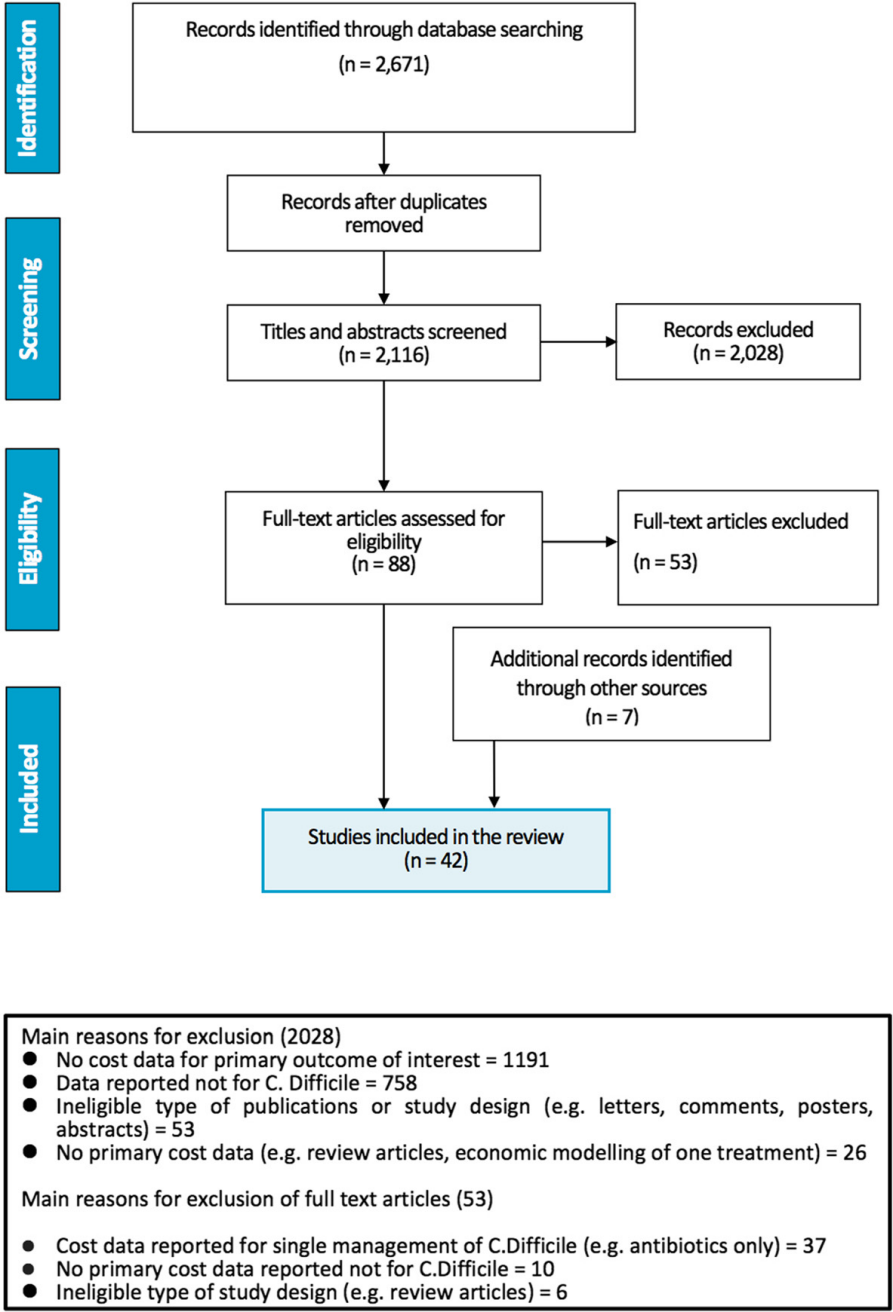
PRISMA diagram of economic burden search of C. difficile

The search strategy identified 2671 references from databases. Seven additional references were identified through other sources. After screening the titles, abstracts and relevant full texts (Fig. 3), a total of 42 studies were included in this review.

### Study characteristics

The characteristics of the 42 included studies [27–68] are summarized in Table 1. Cost data collection periods ranged from 1997 to 2012. Most studies (*n* = 27) used national level databases, with 17 used National Independent Sample (NIS) database and the remaining 10 studies extracted data from various national databases. Fifteen studies were conducted at state level, of which 6 studies only collected data in single hospital. All studies reported cost in hospital level of care, no articles identified in LTCF and community. Nearly all identified references were retrospective hospital database studies (*n* = 40) and only 1 study was a prospective observational study [29] and another study was a decision tree model [48].

**Table 1:** Overview of selected references that assessed economic burden attributable to CDI by type of CDI considered in the US

ID	Reference	State, city	Data collection period	Type of CDI	Population	Sample size (Total)	Sample size (CDI cases)	Age of CDI patients Mean ± SD or (Range), years	CDI definition (short)	Quality assessment	Statistical methodology	Data source
1	Ali 2012 [27]	National	2004–2008	Comp.	Liver transplant	193,714	5159	>18	ICD-9; 008.45 (Primary Diagnosis-PD, Secondary Diagnosis-SD)	Low	No matching; no regression	Nationwide Inpatient Sample (NIS)
2	Ananthakrishnan 2008 [28]	National	2003	Comp.	IBD	124,570	2804	>18 CDI: 73^a^; CDI-IBD: 54^a^	ICD-9; 008.45 (PD)	Medium	No matching; regression	NIS
3	Arora 2011 [29]	Houston	2007–2008	Req.	General	85	85	Horn’s Index Score 1&2: 64 ± 19; Horn’s Index Score 3&4: 65 ± 15	Toxin assay	Low	No matching; no regression	St Luke’s Episcopal Hospital
4	Bajaj 2010 [30]	National	National: 2005 Tertiary: 2002–2006	Both	Cirrhosis	83,230	1165	CDI: 69 ± 20; Cirrhosis-CDI: 61 ± 15	ICD-9; 008.45 (PD, SD)	Medium	No matching; regression	NIS
5	Campbell 2013 [31]	National	2005–2011	Comp.	General	NR	4521	Renal impairment 72.9 ± 13.4; Advanced Age: 78.7 ± 7.4; Cancer/BMT 69.2 ± 14.0; IBD 61.2 ± 18.3; Cabx exposure 61.2 ± 14.8	Toxin assay	High	Matching; regression	Health Facts electronic health record (HER) database
6	Damle 2014 [14]	National	2008–2012	Comp.	Colorectal surgery	84,648	1266	>18 63 ± 17	ICD-9; 008.45 (PD, SD)	Medium	No matching; regression	University Health System Consortium database
7	Dubberke 2008 [33]	Missouri	2003–2003	Both	Non- Surgical	24,691	439	67(18–101) ^a^	Toxin assay	High	Matching; regression	Barnes-Jewish Hospital Electronic record
8	Dubberke 2014 [2, 34, 71]	Missouri	2003–2009	Both	Recurrent CDI	3958	421	>18	Toxin assay or clinical diagnosis for recurrent CDI	High	Matching; regression	Barnes-Jewish Hospital Electronic record
9	Egorova 2015 [35]	National	2000–2011	Comp.	Vascular surgery	NR	2808	68.4	ICD-9; 008.45 (PD, SD)	High	Matching: regression	NIS
10	Flagg 2014 [36]	National	2004–2008	Comp.	Cardiac surgery	349,112	2581	All age band	ICD-9; 008.45 (SD)	High	Matching: regression	NIS
11	Fuller 2009 [37]	Maryland and California	2007–2008 for Maryland 2005–2006 for California	Comp.	General	3760	3760	–	Clinical diagnosis	Medium	No matching; regression	Health Services and Cost Review Commission, Maryland; The Office of State-wide Planning and Development, California
12	Glance 2011 [38]	National	2005–2006	Comp.	Trauma	149,648	768	69(45–82) ^a^	Clinical diagnosis	Medium	No matching; regression	NIS
13	Jiang 2013 [39]	Rhode Islands	2010–2011	Comp.	General	225,999	6053	>18 71.4 ± 15.8	ICD-9; 008.45 (SD)	Medium	Matching; no regression	Rhode Island’s 11 acute-care hospitals
14	Kim 2012 [40]	National	2001–2008	Comp.	Cystectomy	10,856	153	>18 68.49 ± 10.52	ICD-9 ; 008.45 (SD)	Medium	No matching; regression	NIS
15	Kuntz 2012 [41]	Colorado	2005–2008	Comp.	General	3067	3067	All age band, Outpatient 62.8 ± 19.4; Inpatient 69.9 ± 16.3	ICD-9 + toxin assay	Medium	No matching; regression	Kaiser Permanente Colorado and Kaiser Permanente Northwest members
16	Lagu 2014 [42]	Massachusetts, Boston one hospital	2004–2010	Comp.	Sepsis	218,915	2348	70.9 ± 15.1	ICD-9; 008.45 (PD, SD) + toxin assay	Medium	Matching; no regression	Baystate Medical Center (Premier Healthcare Informatics database, a voluntary, fee-supported database)
17	Lameire 2015	National	2002–2009	Comp.	Cardiac surgery	512,217	421,294	>40 CABG 65.4 ± 10.5 VS 66.1 ± 12.3	ICD-9; 008.45 (PD, SD)	Medium	No matching; regression	NIS
18	Lawrence 2007 [44]	Missouri	1997–1999	Both	ICU	1872	76	Primary 68.9 (34–93) Secondary 58.7 (16–91)	Toxin assay	Medium	No matching; regression	A 19-bed medical ICU in a Midwestern tertiary care referral center.
19	Lesperance 2011 [45]	National	2004–2006	Comp.	Elective colonic resections	695,010	10,077	>18 All 69.8; Surgery-CDI 68.7	ICD-9; 008.45 (SD)	Medium	No matching; regression	NIS
20	Lipp 2012 [46]	New York	2007–2008	Comp.	General	4,853,800	3883	>17	ICD-9; 008.45 (SD)	Medium	No matching; regression	- The SPARCS database- acute care non-federal hospitals in New York State
21	Maltenfort 2013 [47]	National	2002–2010	Both	Arthroplasty	NR	NR	All age band	ICD-9; 008.45 (PD, SD)	Low	No matching; no regression	NIS
22	McGlone 2012 [48]	National	2008	Comp.	General	NR	NR	>65	ICD-9; 008.45 (SD)	Low	No matching; no regression	Decision tree model
23	Nguyen 2008 [49]	National	1998–2004	Comp.	IBD	527,187	2372	47.4 ± 0.2	ICD-9; 008.45 (secondary diagnosis)	Medium	No matching; regression	NIS
24	Nylund 2011 [50]	National	1997,2000, 2003,2006	Both	Children	10,495,728	21,274	CDI 9.5 ± 0.07(SEM)	ICD-9; 008.45 (PD, SD)	High	Matching: regression	Healthcare Cost and Utilization Project Kids’Inpatient Database
25	O’Brien 2007 [51]	Massachusetts	1999–2003	Req.	General	3692	1036	Primary 70 ± 17.6; Secondary 70 ± 17.2	ICD-9; 008.45 (PD, SD)	Low	No matching; no regression	Massachusetts hospital discharge data
26	Pakyz 2011 [52]	National	2002–2007	Comp.	General	30,071	10,857	CDI 61 ± 17	ICD-9; 008.45 (SD)	High	Matching; regression	University Health System Consorsoum (UHC)
27	Pant 2012 [53]	National	2009	Both	Organ transplant (OT)	244,955	6451	>18, OT-CDI 58 ± 16 ^a^; CDI-only 73 ± 22 ^a^	ICD-9; 008.45 (PD, SD)	Medium	No matching; regression	NIS
28	Pant 2012 (2) [54]	National	2009	Both	Renal disease	184,139	5151	>18, ESRD + CDI 66 ± 14 CDI ONLY 70 ± 17	ICD-9; 008.45 (PD, SD)	Medium	No matching; regression	NIS
29	Pant 2013 [55]	National	2009	Both	Children with IBD	12,610	447	<20, 15.1 ± 4.1	ICD-9; 008.45 (PD, SD)	Medium	No matching; regression	The Healthcare Cost and Utilization Project Kids’ Inpatient Database (HCUP-KID)
30	Peery 2012 [56]	National	From 2009	Req.	General	110,533	110,533	All age band	ICD-9; 008.45 (PD)	Low	No matching; no regression	National Ambulatory Medical Care Survey (NAMCS) and NIS
31	Quimbo 2013 [57]	National	2005–2010	Comp.	High Risk subgroups	21,177	26,620	>18 67.5 ± 17.6	ICD-9; 008.45 (PD, SD)	High	Matching: regression	HealthCare Integrated Research Database
32	Reed 2008	Pennsylvania	2002–2006	Comp.	High Risk subgroups	9164	524	>17	Hospital acquired CDAD	Low	No matching; no regression	A large academic community hospital
33	Sammons 2013 [59]	National	2006–2011	Both	Children	13,295	4447	1–18 6 (2–13) ^a^	ICD-9; 008.45 (PD, SD) + toxin assay	High	Matching; regression	Free-standing children’s hospitals via the Paediatric Health Information System (PHIS)
34	Singal 2014 [60]	National	2007	Comp.	Cirrhosis	89,673	1444	All age band	ICD-9; 008.45 (PD, SD)	Medium	No matching; regression	NIS
35	Song 2008 [61]	Maryland	2000–2005	Both	General	9025	630	>18 unmatched 57.6 matched 60.3	Toxin assay	High	Matching; regression	The Johns Hopkins hospital
36	Stewart 2011 [62]	National	2007	Both	General	82,214	41,207	All age band, 70	ICD-9; 008.45 (PD, SD)	Medium	Matching; no regression	NIS
37	Tabak 2013 [63]	Pennsylvania	2007–2008	Comp.	General	77,257	255	All 64.8 ± 17.6 CDI 71.1 ± 14.8	Toxin assay	High	Matching; regression	Six Pennsylvania hospitals via a clinical research database
38	VerLee 2012	Michigan	2002–2008	Req.	General	517,413	517,413	All age band	ICD-9; 008.45 (PD)	Low	No matching; no regression	All Michigan acute care hospitals
39	Wang 2011 [65]	Pennsylvania	2005–2008	Both	General	7,227,788	78,273	All age band	ICD-9; 008.45 (PD, SD)	High	Matching; regression	The Pennsylvania Health Care Cost Containment Council (PHC4) database
40	Wilson 2013 [66]	National	2004–2008	Comp.	Ileostomy	13,245	217	All age band	ICD-9; 008.45 (SD)	High	Matching; regression	NIS
41	Zerey 2007 [67]	National	1999–2003	Both	Surgical	1,553,597	8113	All age band 70 ^a^m	ICD-9; 008.45 (PD, SD)	Medium	No matching; regression	NIS
42	Zilberberg 2009 [68]	National	2005	Both	Prolonged acute mechanical ventilation	64,910	3468	>18 66.7 ± 15.9	ICD-9; 008.45 (PD, SD)	Medium	Matching; no regression	NIS

*Abbreviations*: *NR* not reported, *IBD* inflammatory bowel disease, *LOS* length of stay, *ICU* intensive care unit, *retrosp*. retrospective, *Comp*. complicating, *Req*. requiring, both requiring and complicating, *PD* primary diagnosis, *SD* secondary diagnosis

^a^ Median (Range)

Most studies (*n* = 15) investigated economic outcomes in all age inpatients. Three studies reported cost data in children less than 20 years old. The mean/median age of the CDI patient groups ranged from 47.4 to 73.0 years. Other studies investigated complicated CDI in high-risk patient groups, such as those with major surgery (*n* = 16), inflammatory bowel diseases (*n* = 2), liver or renal disease (*n* = 4), elderly (*n* = 2) and ICU patients (*n* = 1). There was 1 study each in non-surgical inpatients, sepsis inpatients and patients with prolonged acute mechanical ventilation. There was 1 study focusing only on recurrent CDI in the general population.

The sample sizes of included studies ranged from 85 to 7,227,788, with a median sample size of 83,939. A total of 28.8 million inpatient hospital-days were analysed, of which 1.31 million inpatient hospital-days were CDI patients. The median sample size of CDI population was 2938.

The methods to identify CDI varied according to the type of CDI that was assessed in the study. CDI cases were identified either with laboratory test, i.e. positive *C. diffcile* toxin assay, or hospital discharge diagnosis of C. difficile (primary and/secondary) from administrative datasets using the International Classifications of diseases, Ninth, Clinical Modification, ICD-9-CM 008.45. Clinical diagnosis was also used in two studies.

CDI was classified in three types: Community-onset CDI (CO-CDI) requiring hospitalization, Hospital-onset CDI (HO-CDI) complicating other diseases, or both CDI (Table 2). Most of included studies considered HO-CDI (*n* = 23) or both CDI types (*n* = 17). Only four studies investigated CO-CDI only. However, subgroup data of CO-CDI is also available in studies that reported both CDI types.

**Table 2:** Classification of CDI Cases by Setting of Acquisition

Case definition	Criteria for classification
CO-CDI	- Discharge code ICD-9-CM 008.45 as Primary diagnosis
HO-CDI	- Discharge code ICD-9-CM 008.45 as secondary diagnosis, without a primary diagnosis of a CDI-related symptom (e.g. diarrhea) - Study population ≥ 48 h of hospitalization - Symptom onset and/or positive laboratory assay at least ≥ 48 h hospitalization
Both CDI	- No distinction of settings of acquisition - Discharge code ICD-9-CM 008.45 in any position

*Abbreviations*: *CO-CDI* community-onset CDI, *HO-CDI* hospital-onset CDI, *ICD-9-CM* The International Classification of Diseases, Ninth Revision, Clinical Modification

### CDI costs and LOS

The mean CDI-attributable costs per case of CO-CDI were $20,085 (Range: $7513–$29,662), lower than HO-CDI $34,149 (Range:$1522–$122,318). HO-CDI showed a wider range within which the additional cost for CDI in the general population ranged from $6893 to $90,202 and in high risk groups ranged from $7332 in congestive heart failure patients to $122,318 in renal impairment patients. The mean CDI-attributable LOS was 5.7 days (Range: 2.1–33.4) for CO-CDI, 7.8 (Range:2.3–21.6) days for HO-CDI, and 13.6 (Range: 2.2–16) days for both groups. Cost data and LOS for individual studies are presented in Tables 3 and 4.

**Table 3:** CDI-attributable costs/charges and CDI-related management costs/charges

Author, Year	Population	Outcome	Statistic	Incremental CDI-attributable cost/charges				CDI-related cost/charges				Note
				Sample size	Attributable cost 2015$	SD or 95 % CI		Sample size	CDI only cost 2015$	SD, 95 % CI or IQR		
CO-CDI Inpatient Cost												
Arora 2011 [29]	General	Cost	Median	85	25,436			85	25,436			
O’Brien 2007 [51]	General	Cost	Mean	4015	14,736			4015	14,736			
Peery 2012 [56]	General	Cost	Median	110,553	7513			110,553	7513			
VeerLee 2012 [64]	General	Charges	Mean	68,686	74,211	120,156		68,686	74,211	120,156		
Kuntz 2012 [41]	General	Cost	Mean	1650	929	4800		1650	929	4800		Outpatient
Kuntz 2012 [41]	General	Cost	Mean	1316	11,877	35,923		1316	11,877	35,923		Inpatient
O’Brien 2007 [51]	General	Cost	Median	1036	7263			1036	7263			PD
VeerLee 2012 [64]	General	Charges	Mean	17,413	27,463	40,484		17,413	27,463	40,484		PD
O’Brien 2007 [51]	General	Cost	Mean	3327	16,946	34,655		3327	16,946			Rehospitalisation
Sammons 2013 [59]	Children	Cost	Mean	2060	19,993	15,973	24,013	2060	19,993	15,973	24,013	Community onset
Ananthakrishnan 2008 [28]	IBD	Charges	Median					44,400	16,864			CDI only
Pant 2013 [55]	IBD	Charges	Mean	12,610	12,761	6868	18,655	447	50,050			CDI only
Bajaj 2010 [30]	Cirrhosis	Charges	Mean					58,220	70,309			CDI only
Quimbo 2013 [57]	CDI History	Cost	Mean	1866	29,662	20,798	42,300	933	51,863	36,641	73,411	CDI only
Total numbers/Weighted Mean				224,617	20,085			314,141	23,322			
HO-CDI Inpatient Cost												
Fuller 2009 [37]	General	Cost	Coefficient	1282	18,466	288		1282	18,466	288		Maryland, SD
Fuller 2009 [37]	General	Cost	Coefficient	2478	29,980	271		2478	29,980	271		California, SD
Lipp 2012 [46]	General	Cost	Mean	3826	32,050			3826	32,050			SD
McGlone 2012 [48]	General	Cost	Median	54,046	10,016	8547	12,055	54,046	10,016	8547	12,055	SD Cost-hospital perspective-6 days LOS
McGlone 2012 [48]	General	Cost	Median	54,046	11,116	9476	13,366	54,046	11,116	9476	13,366	10 days LOS
McGlone 2012 [48]	General	Cost	Median	54,046	12,194	10,146	14,896	54,046	12,194	10,146	14,896	14 days LOS
O’Brien 2007 [51]	General	Cost	Median	2656	6630			2656	6630			SD
VeerLee 2012 [64]	General	Charges	Mean	51,273	90,202	146,767		51,273	90,202	146,767		SD
Jiang 2013 [39]	General	Cost	Median	7264	*11,689*			1211	21,751			
Pakyz 2011 [52]	General	Cost	Mean	30,071	*31,180*			10,857	64,732			Unadjusted
Pakyz 2011 [52]	General	Cost	Median	30,071	*24,456*			10,857	39,598	22,400	88,537	Unadjusted
Pakyz 2011 [52]	General	Cost	Mean	30,071	*31,169*			10,857	64,000	63,541	64,458	Adjusted
Tabak 2013 [63]	General	Cost	Mean	1020	6893	1365	13,617	255	22,992	12,222	42,470	
Campbell 2013 [31]	Age > = 65	Cost	Mean	3064	7536	4302	10,771	3064	48,932	67,727		
Quimbo 2013 [57]	Elderly	Cost	Mean	34,732	45,749	43,279	48,359	10,933	83,004	78,548	87,713	
Sammons 2013 [59]	Children	Cost	Mean	2414	99,012	84,626	113,398	2414	99,012	84,626	113,398	
Ananthakrishnan 2008 [28]	IBD	Charges	Median	80,170	*7655*			2804	24,623			
Ananthakrishnan 2008 [28]	IBD	Charges	Mean	80,170	14,368	9467	19,270	–				
Campbell 2013 [31]	IBD	Cost	Mean	84	1522	−14,932	11,888	84	40,194	44,845		
Quimbo 2013 [57]	IBD	cost	Mean	3618	11,825	9851	14,181	1206	42,035	35,918	49,191	
Ananthakrishnan 2008 [28]	Ulcerative colitis (UC)	Charges	Median					1843	26,750			
Nguyen 2008 [49]	UC	Charges	Mean	43,645	*14,749*			196	43,381			Regression
Ananthakrishnan 2008 [28]	Crohn's disease (CD)	Charges	Median					961	22,738			
Nguyen 2008 [49]	CD	Charges	Mean	73,197	*14,316*			329	41,453			Regression
Reed 2008	Digestive disorders	Charges	Mean	2394	*3670*			320	9076	8068		
Damle 2014 [14]	Colorectal surgery	Cost	Median	84,648	14,644	13,700	15,589	1266	21,309	38,218		–
Kim 2012 [40]	Cystectomy	Cost	Mean	10,856	*25,014*			153	57,379	50,204	64,554	
Lesperance 2011 [45]	Elective colonic resection	Charges	Mean	695,010	*84,899*			10,077	158,401			
Reed 2008	Major bowel procedures	Charges	Mean	1035	*25,476*			45	47,064	31,302		
Wilson 2013 [66]	Ileostomy	Cost	Mean	13,462	*20,272*			217	35,076			
Wilson 2013 [66]	Ileostomy	Cost	Coefficient	13,462	17,513	14,106	20,921					
Egorova 2015 [35]	Vascular surgery	Cost	Median	450,251	14,250			4708	36,847	22,912	62,903	
Flagg 2014 [36]	Cardiac surgery	Cost	Median	5160	*19,524*			2580	213,661			Adjusted
Flagg 2014 [36]	Cardiac surgery	Cost	Median	349,122	*38,320*			2580	72,730			Unadjusted
Lemaire 2015 [43]	Cardiac surgery	Cost	Median	421,294	*35,968*			–	72,685			CABG
Lemaire 2015 [43]	Cardiac surgery	Cost	Median	90,923	*59,696*			–	106,141			VS
Reed 2008	OR procedure for infectious /parasitic diseases	Charges	Mean	449	*7462*			32	35,524	25,498		
Glance 2011 [38]	Trauma	Cost	Median	149,656	*24,131*			768	39,296			
Campbell 2013 [31]	Cabx	Cost	Mean	1641	18,567	10,448	26,687	1641	78,948	99,739		
Quimbo 2013 [57]	Cabx	Cost	Mean	17,716	38,413	35,195	41,922	4429	64,242	59,145	69,780	
Lagu 2014 [42]	Sepsis	Cost	Median	4736	5792	4933	6665	2368	28,576	16,496	50,494	
Reed 2008	Septicaemia	Charges	Mean	1211	*9141*			92	22,378	20,591		
Campbell 2013 [31]	Renal impairment	Cost	Mean	3236	5024	1118	8928	3236	50,586	72,180		
Quimbo 2013 [57]	RI	Cost	Mean	22,132	122,318	111,315	134,405	5533	201,212	183,706	220,386	
Ali 2012 [27]	Liver transplant	Charges	Mean	193,714	*77,361*			5159	158,038			
Singal 2014 [60]	Cirrhosis	Charges	Mean	89,673	*23,310*			1444	47,401			
Reed 2008	Congestive Heart Failure	Charges	Mean	2542	*7332*			35	14,738	13,841		
Quimbo 2013 [57]	Immunocompromised	Cost	Mean	14,344	33,632	30,151	37,516	3586	73,612	66,048	82,041	
Campbell 2013 [31]	Cancer/BMT	Cost	Mean	782	687	−6480	7855	782	48,280	72,605		
Total numbers/Weighted mean				3,020,827	34,149			207,801	49,712			
Dubberke 2014 [2, 34, 71]	Recurrent CDI	Cost	Mean	3958	12,163			3958	11,523	4728	26,167	Total cost difference
Dubberke 2014 [2, 34, 71]	Recurrent CDI	Cost	Mean	3958	12,692	9752	15,919					Adjusted
Song 2008 [61]	General	Cost	Median	1260	*373*			630	30,305			
Stewart 2011 [62]	General	Cost	Mean	82,414	*9670*			41,207	26,790			
Wang 2011 [65]	General	Cost	Median	7,227,788	*4914*			78,273	12,081			
Nylund 2011 [50]	Children	Charges	Median	3565	*15,937*			3565	25,549			1997
Nylund 2011 [50]	Children	Charges	Median	4356	*20,750*			4356	31,858			2000
Nylund 2011 [50]	Children	Charges	Median	5574	*23,627*			5574	33,625	11,348	97,822	2003
Nylund 2011 [50]	Children	Charges	Median	7779	*23,362*			7779	35,444	13,601	110,343	2006
Sammons 2013 [59]	Children	Cost	Mean	698,616	51,304	44,746	57,969	698,616	51,304	44,746	57,969	
Dubberke 2008 [33]	Non-surgical	Cost	Median	24,691	*11,749*			439	20,569			Raw data
Dubberke 2008 [33]	Non-surgical	Charges	Median	24,691	*23,961*			439	42,154			Raw data
Dubberke 2008 [33]	Non-surgical	Cost	Mean	24,691	3173	3078	3815					Linear regression
Dubberke 2008 [33]	Non-surgical	Cost	Median	24,691	4190			342	18,842			Matched cases
Dubberke 2008 [33]	Non-surgical	Cost	Mean	24,691	6520	4910	8381					Linear regression, 180 days
Dubberke 2008 [33]	Non-surgical	Cost	Median	24,691	9284			342	35,414			Matched cases, 180 days
Zerey 2007 [67]	Surgical	Charges	Median	1,553,597	*59,424*			8113	81,708			
Zerey 2007 [67]	Surgical	Charges	Coefficient	1,553,597	94,402	91,589	97,216					Multivariate regression analysis
Zilberberg 2009 [68]	Prolonged acute mechanical ventilation (PAMV)	Cost	Median	64,910	*48,065*			3468	190,188	107,689	333,290	Unadjusted
Zilberberg 2009 [68]	PAMV	Cost	Mean	3370	12,616	9186	16,046	3468	91,039	71,306		Adjusted
Lawrence 2007 [44]	ICU	Cost	Median	1872	*7043*			76	15,016			ICU stay
Lawrence 2007 [44]	ICU	Cost	Median	1872	*36,095*			76	60,723			Entire hospital stay
Bajaj 2010 [30]	Cirrhosis	Charges	Mean	83,230	49,460			1165	96,678			
Maltenfort 2013 [47]	Arthroplasty	Charges	Median	–	43,648			–	84,877	52,498	142,827	
Pant 2012 [53]	Organ transplant	Charges	Mean	49,198	77,246	73,412	81,080	63,651	42,054	69,033		
Pant 2012 (2) [54]	Renal disease	Charges	Coefficient	184,139	69,679	68,338	71,020	59,793	87,982			
Pant 2013 [55]	IBD	Charges	Mean	12,610	39,453	32,470	46,436					
Total numbers/Weighted Mean				10,012,927	14,403			981,005	45,421			

*Abbreviations*: *CO-CDI* community-onset CDI, *HO-CDI* hospital-onset, *PAMV* prolonged acute mechanical ventilation, *Cabx* concomitant antibiotic use, *UC* ulcerative colitis, *CD* Crohn’s disease, *IBD* inflammatory bowel disease, *ICU* intensive care unit, *CABG* coronary artery bypass grafting, *VS* valvular surgery, *BMT*, *PD* primary diagnosis, *SD* secondary diagnosis, *Calculated numbers were marked in Italic, attributable cost = cost of CDI group- cost of control non-CDI group*

**Table 4:** CDI-attributable LOS and CDI-related LOS

Reference	Population	Statistic	CDI VS NO CDI LOS (Days)				CDI LOS (Days)			
			Sample size	Value	SD or 95 % CI		Sample size	Value	SD or 95 % CI	
CO-CDI Inpatient days										
Arora 2011 [29]	Horn’s index 1&2	Mean	33	15.1	16.2		33	15.1	16.2	
Arora 2011 [29]	Horn’s index 3&4	Mean	52	33.4	33.3		52	33.4	33.3	
Kuntz 2012 [41]	General outpatient	Mean	1650	10.0	17.0		1650	10.0	17.0	
Kuntz 2012 [41]	General inpatient	Mean	1316	14.9	20.9		1316	14.9	20.9	
O’Brien 2007 [51]	General	Mean	4015	6.4			4015	6.4		
Pant 2013 [55]	IBD	Coefficient	12,610	2.1	1.4	2.8		2.1	1.4	2.8
Peery 2012 [56]	General	Median	110,553	5.0			110,553	5.0		
Quimbo 2013 [57]	CDAD History	Mean	1866	2.9	2.4	3.6	933	8.9	7.2	11.0
Sammons 2013 [59]	Children	Median	2060	5.6	4.5	6.6	2060	6.0	4.0^a^	13.0^a^
VeerLee 2012 [64]	General	Mean	68,686	7.1	7.0		68,686	7.1	7.0	
Weighted Mean			202,841	5.7			189,298	5.9		
HO-CDI inpatient days										
Jiang 2013 [39]	General	Median	7264	*8.0*			1211	13.0		
Lipp 2012 [46]	General	Mean	3826	12.0			3826	12.0		
Pakyz 2011 [52]	General	Mean	30,071	11.1			10,857	21.1	21.0	21.2
Tabak 2013 [63]	General	Median	1020	2.3	0.9	3.8	255	12.0	9.0^a^	21.0^a^
Wang 2013	General	Median	7,227,788	*7.0*			78,273	6.0	4.0^a^	11.0^a^
Campbell 2013 [31]	Age > = 65	Mean	3064	3.0	1.4	4.6	3064	21.3	25.3	
Quimbo 2013 [57]	Elderly	Mean	34,732	7.8	7.5	8.1	10,933	18.8	18.2	19.5
Sammons 2013 [59]	Children	Median	2414	21.6	19.3	23.9	2414	23.0	12.0^a^	44.0^a^
Ananthakrishnan 2008 [28]	IBD	Median	80,170	3.0			2804	7.0		
Campbell 2013 [31]	IBD	Mean	84	3.0	−2.3	8.3	84	21.0	19.1	
Quimbo 2013 [57]	IBD	Mean	3618	3.3	2.9	3.7	1206	12.8	11.6	14.2
Nguyen 2008 [49]	Crohn’s disease	Mean	73,197	*3.8*			329	9.5		
Nguyen 2008 [49]	Ulcerative colitis	Mean	43,645	*3.2*			196	9.9		
Reed 2008	Digestive disorders	Mean	2394	*3.0*			320	6.9	5.2	
Damle 2014 [14]	Colorectal surgery	Median	84,648	8.4	8.0	8.9	1266	13.0	18.0	
Lesperance 2011 [45]	Elective colonic resection	Mean	695,010	*11.7*			10,077	22.6		
Reed 2008	Major bowel procedures	Mean	1035	*10.0*			45	20.9	11.3	
Wilson 2013 [66]	Ileostomy	Mean	13,462	11.6			217	18.7		
Campbell 2013 [31]	Cabx exposure	Mean	1641	7.8	5.7	9.9	1641	29.3	34.7	
Quimbo 2013 [57]	Concomitant Antibiotic Use	Mean	17,716	7.8	7.4	8.3	4429	17.9	17.0	18.9
Lagu 2014 [42]	Sepsis	Mean	4736	5.1	4.4	5.7	2368	19.2		
Reed 2008	Septicemia	Mean	1211	5.0			92	10.7	7.6	
Egorova 2015 [35]	Vascular surgery	Median	450,251	*6.7*			4708	15.0	9.0^a^	25.0^a^
Flagg 2014 [36]	Cardiac surgery	Median	349,122	*10.0*			2580	21.0		
Glance 2011 [38]	Trauma	Median	149,656	*10.0*			768	16.0		
Lemaire 2015 [43]	Cardiac surgery (CABG)	Median	421,294	*12.0*				19.0		
Lemaire 2015 [43]	Cardiac surgery (VS)	Median	90,923	*16.0*				24.0		
Reed 2008	Congestive Heart Failure	Mean	2542	*5.0*			35	9.7	7.0	
Reed 2008	OR procedure for infectious /parasitic diseases	Mean	449	*2.0*			32	14.7	8.6	
Lawrence 2007 [44]	ICU	Median					76	14.9	1.0^b^	86.0^b^
Lawrence 2007 [44]	ICU	Median					76	38.3	4.0^b^	184.0^b^
Ali 2012 [27]	Liver transplant	Mean	193,714	*10.1*			5159	17.8		
Singal 2014 [60]	Cirrhosis	Mean	89,673	*7.5*			1444	13.9		
Quimbo 2013 [57]	Immunocompromised	Mean	14,344	8.4	7.9	9.0	3586	22.1	20.6	23.7
Campbell 2013 [31]	Renal impairment	Mean	3236	4.0	2.9	5.1	3236	22.7	28.2	
Quimbo 2013 [57]	Renal impairment	Mean	22,132	17.3	16.4	18.3	5533	37.5	35.5	39.6
Campbell 2013 [31]	Cancer/BMT	Mean	782	4.0	2.3	5.7	782	21.3	18.5	
Weighted Mean			10,120,864	7.8			168,892	13.5		
Both CO-CDI and HO-CDI inpatient cost										
Song 2008 [61]	General	Median	1260	*4.0*			630	22.0		
Stewart 2011 [62]	General	Mean	82,414	*5.1*			41,207	13.0	14.0	
Nylund 2011 [50]	Children, 1997	Median	3565	3.0			3565	5.0	3.0^a^	14.0^a^
Nylund 2011 [50]	Children, 2000	Median	4356	4.0			4356	6.0	3.0^a^	15.0^a^
Nylund 2011 [50]	Children, 2003	Median	5574	4.0			5574	6.0	3.0^a^	14.0^a^
Nylund 2011 [50]	Children, 2006	Median	7779	4.0			7779	6.0	3.0^a^	15.0^a^
Sammons 2013 [59]	Children	Median	698,616	12.2	10.6	13.8	698,616	10.0	5.0^a^	23.0^a^
Bajaj 2010 [30]	Cirrhosis	Mean	83,230	7.1			1165	14.4		
Bajaj 2010 [30]	CDI only	Mean					58,220	12.7		
Pant 2013 [55]	IBD	Mean	12,610	2.2	1.5	2.8	447	8.2		
Dubberke 2008 [33]	Non-surgical	Median	24,691	*6.0*			439	10.0	2. 0^b^	87.0^b^
Lawrence 2007 [44]	ICU stay	Median	1872	*3.1*			76	6.1	1.0^b^	86.0^b^
Lawrence 2007 [44]	Hospital stay	Median	1872	*14.4*			76	24.5	2.0^b^	184.0^b^
Maltenfort 2013 [47]	Arthroplasty	Median	–	7.0			–	10.0	7.0^a^	17.0^a^
Zerey 2007 [67]	Surgical	Median	1,553,597	16.0	15.6	16.4	8113	18.0		
Pant 2012 [53]	Organ transplant	Median	49,198	9.6	9.3	9.9	63,651			
Pant 2012 (2) [54]	Renal disease	Coefficient	184,139	9.4	9.2	9.5	59,793			
Zilberberg 2009 [68]	Prolonged acute mechanical ventilation	Median	3370	6.1	4.9	7.4	3468	25.0	15.0^a^	40.0^a^
Weighted Mean			2,718,143	13.6			957,175	9.0		

*Abbreviations*: *CO-CDI* community-onset CDI, *HO-CDI* Hospital-onset CDI, *PAMV* prolonged acute mechanical ventilation, *Cabx* concomitant antibiotic use, *UC* ulcerative colitis, *CD* Crohn’s disease, *IBD* inflammatory bowel disease, *ICU* intensive care unit, *CABG* coronary artery bypass grafting, *VS* valvular surgery, *BMT*, *PD* primary diagnosis, *SD* secondary diagnosis, *Calculated numbers were marked in Italic, attributable cost = cost of CDI group- cost of control non-CDI group*

^a^Q1-Q3

^b^Min-Max

Using a Monte Carlo simulation, we generated point estimates and 90 % CI for both cost and LOS; the meta-analysis results are shown in Table 5. The total cost of inpatient management of CDI-related disease was $42,316 (90 % CI: $39,886–$44,765) per case, of which the total CDI-attributable cost was $21,448 (90 % CI: 21,152–21,744) per case. For the inpatient management, the attributable cost for those HO-CDI was $34,157 (90 % CI: $33,134–$35,180), which was 1.5 times as much as CO-CDI management $20,095 (90 % CI: $4991–$35,204).

**Table 5:** Meta analysis results of cost and LOS of CDI management

CDI category	CDI-attributable cost per case (2015 US$)			CDI-related cost per case (2015 US$)			CDI-attributable LOS per case (Days)			CDI-related LOS per case (Days)		
	Weighted mean	90 % CI		Weighted mean	90 %CI		Weighted mean	90 % CI		Weighted mean	90 % CI	
CO-CDI	20,095	4991	35,204	23,329	12,520	34,141	5.7	4.1	7.3	5.7	4.1	7.3
HO-CDI	34,157	33,134	35,180	53,487	42,054	66,326	9.7	9.7	9.7	14.1	13.0	15.4
Both CO-CDI and HO-CDI	17,650	17,292	18,009	46,000	42,502	49,533	10.4	9.7	11.0	11.8	7.1	17.6
Overall inpatient	21,448	21,152	21,744	42,316	39,886	44,765	9.7	9.6	9.8	11.1	8.7	13.6

*Abbreviations*: *CO-CDI* community-onset CDI, *HO-CDI* Hospital-onset CDI

Similar patterns were observed in LOS data. The total CDI-related LOS was 11.1 days (90 % CI: 8.7–13.6) and CDI-attributable LOS was 9.7 (90 % CI: 9.6–9.8). The HO-CDI patients had longer CDI-attributable LOS 9.7 days (90 % CI: 9.7–9.7) than CO-CDI patients 5.7 days (90 % CI: 4.1–7.3).

### CDI annual national impact estimate

The total burden of healthcare facility CDI in US was estimated 293,300 (Range: 264,200–453,000) cases per year [25]. The total financial burden of CDI inpatient management was estimated to be US$6.3 (Range: $1.9–$7.0) billion in 2015, which required 2.4 million days of hospital stay. The total CDI related disease management cost was nearly doubled at US$12.4 (Range: $3.7–$14.4) billion in 2015 (Table 6). A sensitivity analysis showed that the total CDI-attributable cost ranged from $1.31 to $13.61, which covers our estimates (Additional file 1).

**Table 6:** Total cost of CDI management in US

Total number of HCF CDI cases per year (2011) [25]	Mean	95 % CI	
All population ≥2 years Median	293,300	264,200	322,500
Adults ≥18 Upper boundary	288,900	261,100	316,700
Adults ≥18 Lower boundary	133,887	91,780	195,402
Cost per CDI case management (2015 US$)	Weighted Mean	90 % CI	
Overall CDI-attributable cost	21,448	21,152	21,744
Overall CDI-related cost	42,316	39,886	44,765
Total cost per year (in Billions, 2015 US$)	Weighted Mean	Range	
Total CDI-attributable cost per year	6.29	1.94	7.01
Mean	6.29	5.59	7.01
Upper boundary	6.19	5.52	6.88
Lower boundary	2.87	1.94	4.25
Total CDI-related cost per year	12.41	3.66	14.44
Mean	12.41	5.59	14.44
Upper boundary	12.25	10.41	14.18
Lower boundary	5.67	3.66	8.75

*Abbreviations*: *HCF* healthcare facility, *CDI* clostridium difficile infection, *CI* confidence intervals

### Quality assessment

A summary of the quality assessment for statistical methods in included studies is shown in Additional file 1. There were 13 studies of high quality, 21 studies with medium quality and 8 low quality studies.

## Discussion

We systematically reviewed 42 published cost studies of CDI case management in the past 10 years (2005–2015) and found a significant financial burden associated with CDI in the US. The total CDI-attributable cost was US$6.3 billion, which is higher than previously reported (range US$1.1–4.8 billion) [14, 16, 17]. The mean cost for CDI-attributable hospitalized patients per case was US$21,448, nearly half of the mean CDI-related inpatient cost.

This review facilitated a meta-analysis of a large number of cost studies for costs related to CDI management and provided an uncertainty range. Zimlichman et al [17] applied this method to calculate CDI cost based on cost data from two cost-of-illness studies (O’Brian 2007 [51] & Kyne 2002 [69]) and obtained a lower cost [2012US $11,285 ($9118–$13,574)] than ours. Our review combined 100-point estimates and ranges from 42 individual studies, which provided more accurate and comprehensive data of the cost result. Despite the methodological heterogeneity in perspectives, treatment procedure and statistical analysis, each included study met our inclusion criteria, which were defined to identify studies that provided real world estimates of costs, therefore the combination of these data with uncertainty range represented a valuable and reliable summary of CDI-related cost.

Furthermore, we evaluated hospital onset CDI and community onset CDI separately. We found that CDI complicating hospitalization cost more than CDI requiring hospitalization and the former had longer attributable hospital stay. Therefore, other factors, such as comorbidity, may contribute to infections and increase the difficulty of CDI treatment.

We estimated that the total cost attributable to CDI management in the US was nearly US$6.3 (Range: $1.9–$7.0) billion, which is similar to Dubberke and Olsen’s estimates at $4.8 billion [14], but significantly higher than other studies (US$ 1.5 billion in Zimlichman et al [17] and $1.1 billion in Ghantoji et al [16]). The later studies reported lower attributable cost per case based on a limited number of studies before 2005, which arguably is out-of-date. To compare with the latest review on global CDI cost (Nanwa et al [26]), this review identified 8 additional studies with recent data. Nanwa et al [26] found that the mean attributable CDI costs ranged from US$8911 to US$30,049, which is similar to our results.

In this study, we only assessed the quality of study emphasizing statistical methods and did not use the modified economic evaluation guideline as other COI systematic reviews. Cost and LOS estimation of healthcare-associated infections has the potential to be misleading if the confounders such as patients’ comorbidities or daily severity of illness were not properly controlled for. Using either the matching design or multivariable regression analysis allows to control known confounders and may, in part, address selection bias [70]. We found that whether advanced statistical methods were used and described was crucial for the assessment of data quality, which has not be fully captured by the existing quality assessment tool. Therefore in this study we assessed quality of included studies using this new method. Moreover, Nanwa et al [26] has evaluated the methodological completeness of most included studies (34 out of 42); we agree with their recommendations regarding possible improvement of future cost-of-illness study. However, we need to bear in mind that cost effects or excess LOS are still likely to be overestimated if the interval to onset of HAI is not properly accounted for in the study design or analysis [70].

Our systematic review has some limitations. First, all included studies reported direct medical costs from hospital perspective, therefore indirect cost to patients and society and costs of additional care after hospital discharge, have not been captured. No studies reported indirect cost (productivity loss due to work day losses) of patients or care-givers, and we failed to identify studies assessing cost of CDI in long-term care facilities, where about 9 % of CDI patients were discharged to for an average of 24 days of after-care. This would result in an additional US$141 million burden on the healthcare system and society due to LTCF transfers [14]. Second, we did not separate primary CDI from recurrent CDI cost in our review because only two studies reported cost specifically to recurrent CDI $12,592 (Range: $9752, $15,919) [2]. Moreover, we found it difficult to exactly match the CDI case definition in cost study (e.g. ICD10 Code primary diagnosis and secondary diagnosis) with the case definition in epidemiology studies (e.g. community onset, hospital onset), therefore we did not estimate CDI patients managed at outpatient and community settings due to lack of both epidemiology and economic data. The total costs of CDI management may be higher than our current estimate. Fourth, unlike other published reviews, we did not include cost studies from countries other than the US nor facilitate any international comparison. This study initially aimed to identify cost-of-illness studies in North America, but we did not find any studies reporting cost data from Canada. This is likely because we restricted our search to English language databases. Therefore the cost of CDI management in Canada remains unknown. However, we did not apply any language restrictions to the current review.

Effective prevention can reduce the burden of diseases. Strategies have been promoted such as appropriate use of antimicrobials, use of contact precautions and protective personal equipment to care for infected patients, effective cleaning and disinfection of equipment and the environment, and early recognition of disease as primary prophylaxis [71]. As CDI is an infectious disease, the population at risk would benefit from an effective vaccine, which is currently under development [72, 73].

More cost of illness studies for recurrent CDI, or in LTCF, and indirect cost from a societal perspective are needed in the future. We would also recommend that published studies report their methods and include point estimates with uncertainty range. Further economic studies for CDI preventive interventions are needed.

## Conclusion

This review indicates that CDI places a significant financial burden on the US healthcare system. In addition, our findings suggest that the economic burden of CDI is greater than previously reported in the US. This review provides strong evidence to aid policy-making on adequate resource allocation to CDI prevention and treatment in US.

## Additional files

Additional file 1: Appendices-cdiff cost review.docx; Addpendix 1–5; Appendix 1. Embase and Medline searches for each topic of interest (13th July 2015) , Appendix 2. Inclusion and exclusion criteria, Appendix 3. Statistical methods used in selected studies and quality assessment Appendix, 4. Total number of CDI cases in United States 2011, Appendix 5. Sensitivity analysis results (DOCX 101 kb) Additional file 2: CDI Cost Review.xlsx; CDI cost review; CDI cost review data extraction primary results (XLSX 529 kb)

## Abbreviations

CDI: *clostridium difficile* infection
CIs: confidence intervals
CO CDI: community-onset CDI
HCF: healthcare facility
HIV: human immunodeficiency virus
HO-CDI: hospital-onset cdi
ICD-9-CM: the international classification of diseases, ninth revision, clinical modification
ICUs: intensive care units
IQR: interquantile range
LTCF: long-term care facility
NIS: national independent sample
SD: standard deviation
US: United States
