# Supplementary material for: Cost of hospital management of Clostridium difficile infection in United States—a meta-analysis and modelling study
Source: BMC Infect Dis. 2016 Aug 25;16(1):447. doi: 10.1186/s12879-016-1786-6 (PMC5000548; doi:10.1186/s12879-016-1786-6)
Supplement: Additional file 1: — Appendices-cdiff cost review.docx; Addpendix 1–5; Appendix 1. Embase and Medline searches for each topic of interest (13th July 2015) , Appendix 2. Inclusion and exclusion criteria, Appendix 3. Statistical methods used in selected studies and quality assessment Appendix, 4. Total number of CDI cases in United States 2011, Appendix 5. Sensitivity analysis results (DOCX 101 kb) [file 12879_2016_1786_MOESM1_ESM.docx]

# Appendices

Appendix 1. Embase and Medline searches for each topic of interest (13^th^ July 2015)

| Topic of interest | #Search | Key words | #references |
| --- | --- | --- | --- |
| Economic burden of CDI (Medline) | 1 | exp *economics/ | 513，251 |
|  | 2 | exp *costs and cost analysis/ | 191,444 |
|  | 3 | exp *health care costs/ | 50,046 |
|  | 4 | (cost$ or economic$ or expenditure$ or price$ or pharmaconomic$).tw. | 503,200 |
|  | 5 | hospitalization.sh. | 77075 |
|  | 6 | length of stay.sh. | 63469 |
|  | 7 | patient discharge.sh. | 20,416 |
|  | 8 | or/ 1-7 | 984095 |
|  | 9 | clostridium difficile.ti,ab. | 8664 |
|  | 10 | exp Clostridium difficile/ | 6301 |
|  | 11 | or/9-10 | 9360 |
|  | 12 | 8 and 11 | 890 |
|  | 13 | limit 12 to yr="2005 -Current" | 685 |
| Economic burden of CDI (EMBASE) | 1 | exp *economics/ | 229,441 |
|  | 2 | exp "cost"/ or "cost benefit analysis"/ or exp "cost effectiveness analysis"/ | 390237 |
|  | 3 | health/ or exp "health care cost"/ | 392145 |
|  | 4 | (cost$ or economic$ or expenditure$ or price$ or pharmaconomic$).tw. | 712229 |
|  | 5 | hospitalization.sh. | 230048 |
|  | 6 | length of stay.sh. | 100769 |
|  | 7 | or/ 1-6 | 1433557 |
|  | 8 | clostridium difficile.ab,ti. | 12381 |
|  | 9 | exp Peptoclostridium difficile/ | 738 |
|  | 10 | 8 or 9 | 12516 |
|  | 11 | 7 and 10 | 2235 |
|  | 12 | limit 12 to yr="2005 -Current" | 1986 |

kf: Keyword heading word; sh: MeSH Subject Heading; hw: Subject heading word; ab: abstract; af: all fields; ti,ab: title or abstract; tw: text word

Appendix 2. Inclusion and exclusion criteria

| Inclusion/Exclusion elements | Inclusion criteria | Exclusion criteria |
| --- | --- | --- |
| Population | Age: Any  Gender: Any  Race: Any  Disease:  • Clostridium difficile (C. difficile) infections (CDI)  • C. difficile-associated diarrhea (CDAD)  • Hospital induced diarrhea  Disease severity: Any  Comorbidity: Any  Geographical area: USA, Canada. | Geographical area: Countries not listed in the inclusion criteria |
| Interventions | Any intervention given for the treatment of CDI or diagnostic test or management program | None |
| Comparators | Any comparator | None |
| Outcomes of  interest | Economic burden of CDI: treatment costs, inpatient costs (hospitalisation, isolation , ICU, LOS, discharge), specific exams (test for diagnosis, MRI, CT-scan), surgery performed for CDI management, blood transfusion, any other interventions directly attributable to CDI, outpatient costs, costs in relation to long term care facility, etc.. | Economic burden of CDI: only costs not attributable to CDI, indirect costs |
| Study designs | Observational studies: prospective cohorts, retrospective chart reviews  Case-control studies  Registries  Database analyses: insurance databases, electronic medical records  Cross-sectional surveys  RCTs | None |
| Study objective | None | Economic burden of CDI: studies with no mention of costs or resource utilisation |
| Publication  timeframe | 10 years for now | Described in the inclusion criteria |

Appendix 3. Statistical methods used in selected studies and quality assessment

|  | **Regression - adjustment** | | **No regression - no adjustment** | |
| --- | --- | --- | --- | --- |
| **Matching** | **HIGH** | **13 studies**  Campbell 2013, Dubberke 2008, Dubberke 2014, Egorova 2015, Flagg 2014, Nylund 2011, ,Pakyz 2011, Quimbo 2013, Sammons 2013, Song 2008, Tabak 2013, Wang 2011, Wilson 2013 | **MEDIUM** | **4 studies**  Jiang 2013, Lagu 2014, Stewart 2011, Zilberberg 2009 |
| **No matching** | **MEDIUM** | **17 studies**  Ananthakrishnan 2008, Bajaj 2010, Damle 2014, Fuller 2009, Glance 2011, Kim 2012, Lameire 2015, Lawrence 2007, Lesperance 2011, Lipp 2012, Nguyen 2008, Pakyz 2011,  Pant 2012, Pant 2012 (2), Pant 2013, Singal 2014, Zerey 2007 | **LOW** | **8 studies (***)  Ali 2012, Arora 2011, Maltenfort 2013, McGlone 2012, O’Brien 2007, Peery 2012, Reed 2008, VerLee 2012 |

**(***) 8 low quality studies were not excluded because type of CDI assessed was ‘requiring’, economic outcomes assessed are thus attributable to CDI

| **United States (USA) CDI cases 2011** | | | | | | | | | |
| --- | --- | --- | --- | --- | --- | --- | --- | --- | --- |
|  |  | **Total-CDI** | | **HCF CDI** | | **HO-HCF CDI** | | **CA-CDI** | |
|  |  | N | CDI Cases (95%CI) | N | CDI Cases (95%CI) | N | CDI Cases (95%CI) | N | CDI Cases (95%CI) |
| Adults ≥18 | Upper boundary (2011) |  | **436,100** (383,900-487,700) |  | **288,900** (261,000-316,700) |  | NR |  | **147,200** (122,900-171,000) |
|  | Lower boundary (2011) | 9 | **213,824** (171,059-267,115) | 6 | **133,887** (91,780-195,402) | 8 | **124,018** (84,871-181,914) | 5 | **18,750** (8,552-41,448) |
| ≥2 years | Upper boundary (2011) |  | **453,000**  (397,100-508,500) |  | **293,300 (264,200-322,500)** |  | **107,600** (97,200-118,000) |  | 159,700 (132,900-186,000) |
|  | Lower boundary (2011) | 3 | **147,481** (105,295-206,130) | 0 | NA | 4 | **84,030** (25,723-271,983) | 0 | NA |
|  | CDC report (No year) | "at least" | 250,000 illnesses |  |  |  |  |  |  |
|  | Point prevalence (2011) |  |  | Magill | **80,400**  (23,700-155,00) |  |  |  |  |
| N: Number of studies included in meta-analysis; CI: Confidence Intervals; Upper boundary Lessa et al (2015); lower boundary: systematic review and meta-estimates using HCUP 2011 hospital admissions data (3,289,600 adults; 34,298,000 ≥1 years of age) | | | | | | | | | |

Appendix 4. Total number of CDI cases in United States 2011

| Classification | Case definitions (from Lessa 2015) |
| --- | --- |
| CA-CDI | On the basis of the initial medical review, a case was classified as community-associated if the C. difficile–positive specimen was collected on an outpatient basis or within 3 days after hospital admission and the patient had no documented overnight stay in a health care facility during the previous 12 weeks. |
| HCF | All other cases, which can be further categorised into three mutually exclusive groups:   - **community onset** associated with a health care facility   Cases with C. difficile-positive specimen collected in an outpatient setting or ≤ 3 calendar days after hospital admission from a private residence and documented overnight stay in a healthcare-facility (i.e., hospital or nursing home) in the prior 12 weeks.   - **hospital onset**   Cases with C. difficile-positive specimen collected >3 calendar days after hospital admission or in a long-term acute care hospital.   - **nursing home onset**   Cases with C. difficile-positive specimen collected in a nursing home or from a nursing home resident either in an outpatient setting or within three days after hospital admission. |

Appendix 5. Sensitivity analysis results

| Total Number of HCF CDI Cases per year (2011)^25^ | Mean | | 95%CI | |
| --- | --- | --- | --- | --- |
| Scenario 1: All population ≥2 years Median | | 250,000 | 147,481 | 453,000 |
| Cost per CDI case management (2015 US$) | | **Weighted Mean** | **90%CI** | |
| Overall CDI-attributable cost | | 21,448 | 21,152 | 21,744 |
| Scenario 2:CDI-attributable cost (Nanwa 2015^26^) | | 19,480 | 8,911 | 30,049 |
| Scenario 3:CDI-attributable cost (Zimlichman 2013^17^) | | 11,666 | 9,425 | 14,032 |
| Overall CDI-related cost | | 42,316 | 39,886 | 44,765 |
| Total cost per year (in Billions, 2015 US$) | | **Weighted Mean** | **Range** | |
| Total CDI-attributable cost per year | |  |  |  |
| Scenario 1 | | 6.08 | 3.28 | 9.71 |
| Scenario 1&2 | | 4.87 | 1.31 | 13.61 |
| Scenario 1&3 | | 2.91 | 1.39 | 6.36 |
| Total CDI-related cost per year Scenario 1 | | 12.00 | 6.47 | 19.17 |
